# Surgical laser therapy for cryptoglandular anal fistula: Protocol of a systematic review and meta-analysis

**DOI:** 10.1371/journal.pone.0279388

**Published:** 2023-01-04

**Authors:** Zubing Mei, Zhijun Zhang, Ye Han, Peixin Du, Wei Yang, Qingming Wang, De Zheng

**Affiliations:** 1 Department of Anorectal Surgery, Shuguang Hospital, Shanghai University of Traditional Chinese Medicine, Shanghai, China; 2 Anorectal Disease Institute of Shuguang Hospital, Shanghai, China; Universidade Federal dos Vales do Jequitinhonha e Mucuri, BRAZIL

## Abstract

**Introduction:**

Anal fistula is the natural evolution of perianal abscess and one of the most common perianal diseases for adults. For complex fistula, it is still very challenging for anorectal surgeons to manage. With the introduction of laser technique in surgery, it is becoming more and more widely used for the treatment of cryptoglandular anal fistula. During the past decade, numerous studies have reported the clinical effectiveness and postoperative outcomes of different forms of laser treatment for anal fistula. However, as these studies were varied in terms of baseline characteristics, the evidence for the true clinical effectiveness of laser treatment for anal fistula need further critical appraisal. Therefore, the purpose of this study is to evaluate the outcomes of surgical laser therapy for cryptoglandular anal fistula stratified by laser type and Parks’ classification through a synthesis of quantitative and qualitative evidence.

**Methods and analysis:**

This study will be carried out with adherence to the Cochrane Handbook. We will search PubMed, Cochrane Library, and Embase until June, 2022 to identify all relevant interventional and observational studies examining the effects of laser therapy on the clinical outcomes for cryptoglandular anal fistula. Data extraction from eligible studies will be performed independently by two unblinded authors using standardized extraction forms. Risk of bias assessment for each study will be conducted using Cochrane tool for randomized controlled trials (RCTs) and the Newcastle–Ottawa scale (NOS) tool for observational studies. The DerSimonian-Laird random-effects model will be used to calculate the pooled estimates. Heterogeneity will be examined by subgroup analysis stratified by laser type and Parks’ classification and other study characteristics. Potential publication bias will be assessed by funnel plot symmetrical and Egger’s regression tests.

**Conclusions:**

The synthesis of quantitative and qualitative evidence of this systemic review will yield updated and comprehensive evidence of laser treatment on specific outcomes, which can provide anorectal surgeons with high level evidence-based recommendations to improve patient care and clinical outcomes. **OSF registration number:** DOI 10.17605/OSF.IO/36ADW.

## Introduction

Anal fistula, also known as fistula-in-ano, is the natural progression of perianal abscess. In fact, the disease begins with suppurative inflammation of the anal crypts, and if left untreated, the inflammation can spread to the adjacent anorectal spaces, including pelvirectal space, retrorectal space, ischiorectal space, perianal space and transsphincter space [1]. At present, the theoretical basis for the pathogenesis of cryptoglandular perianal fistula is still controversial. According to the cryptoglandular theory of perianal fistulas, they develop from the rectal glands, originate from the intersphincteric plane and perforate the internal sphincter. Rectal glands involved in the inflammatory process (including secondary pro-inflammatory factors) may play a significant role in the development of cryptoglandular anal fistula [2]. The two main goals of anal fistula treatment are successful healing and preservation of the anal sphincter. Complex anal fistulas remain extremely difficult to treat in the field of anal surgery [3–6]. Although there are a number of surgical treatment options available, such as ligation of the intersphincteric fistula tract (LIFT), video-assisted anal fistula treatment, adipose-derived stem cells, endorectal advancement flap, anal fistula plug and fibrin glue [7–11], their use is severely limited due to the high recurrence rate and the influence of sphincter function. The long-term effect and success rate of these surgical approaches are not as satisfactory as the traditional surgical method fistulotomy for simple anal fistula [12,13].

Laser, as a newly surgical technique, has grown in popularity in recent years, particularly for the treatment of benign perianal diseases such as anal fistula [14]. The laser fiber was first introduced to fistula surgery due to its good tissue penetration, and the laser beams were used to destroy and wipe out the fistula. One of its advantages is that it can be used to diagnose and treat disease in confined spaces [1,15]. Another significant advantage is that low laser power can coagulate diseased tissue without vaporizing it [16]. Photodynamic therapy (PDT) [17,18] is another application of laser that is based on the principle that laser causes tissue necrosis by activating photosensitizer without increasing tissue temperature [16]. During fistula laser closure, the laser fiber is introduced from the outside of the fistula and precisely reaches the lesion site. The laser energy can shrink the surrounding tissue, causing protein denaturation. Its advantages include ease of operation, less invasion, faster recovery, and fewer postoperative complications.

Numerous studies, as well as a few quantitative or qualitative analyses have reported on the clinical efficacy and postoperative outcomes of various types of laser treatment for anal fistula [19–22]. However, because these studies differed in terms of study design, sample size, Parks’ Classification for anal fistula patients, laser treatment patterns, and reported outcomes, the evidence for the true clinical effectiveness of laser treatment for anal fistula requires further critical appraisal. As a result, we will critically evaluate the postoperative outcomes for laser treatment of anal fistula using a synthesis of quantitative and qualitative evidence stratified by both laser treatment patterns and Parks’ Classification.

## Methods and analysis

### Protocol registration

We will perform and report this qualitative evidence synthesis protocol according to the Preferred Reporting Items for Systematic Reviews and Meta-Analyses Protocols (PRISMA-P) guidelines [23] (S1 Table) and the Cochrane Handbook (http://handbook.cochrane.org). The registered protocol of this study can be found at the Open Science Framework (OSF) website (https://osf.io/36adw) with the registration number of DOI 10.17605/OSF.IO/36ADW.

### Data sources and search strategies

The following three major databases (PubMed, Embase, and Cochrane Library,) will be systematically searched from inception through June, 2022 by adapting specific search strategies which are presented in details in Table 1. These studies will be identified if they have interventional or observational study design examining the outcomes or effect of laser ablation for cryptoglandular anal fistula. We will apply Medical Subject Heading (MeSH) terms or EMBASE Subject Headings (Emtree) terms along with the corresponding free text words that are pertaining to perianal fistula, laser ablation, and outcomes. The following keywords will be used to identify relevant studies: including terms on anal fistula ((anus OR anal OR anorectal OR perianal) AND (fistula OR fistulotomy OR fistulectomy)) AND laser therapy (laser OR fistula laser closure OR FiLaC) AND treatment outcomes (outcomes OR complication OR reoperation OR continence OR manometry OR healing OR recurrence OR dehiscence). These search terms will be combined with the Boolean operators OR and AND. Reference lists of included studies will be hand searched to identify additional relevant work. Furthermore, we will also conduct manual search of abstracts from annual scientific meeting of the American Society of Colon and Rectal Surgeons (ASCRS), European Society of Coloproctology (ESCP) and Asia Pacific Federation of Coloproctology (APFCP). For duplicate data in more than one publication, we will select the study with the largest sample. Search results will be imported into Endnote X9 software (Thomson Reuters for Windows version) to check for duplicate entries which will be removed accordingly. For duplicate records that cannot be recognized by the Endnote software, we will manually remove them by comparing publication authors, titles and publication dates. Two authors will independently conduct the literature search, screen of titles and abstracts, selection of included studies and data extraction. A third author will resolve discrepancies by discussion and adjudication who will examine the studies independently.

**Table 1:** Search strategy for Pubmed database.

***Anal fistula terms*:**
1 "Rectal Fistula"[Mesh]
2 ((anus OR anal OR anorectal OR perianal) AND (fistula OR fistulotomy OR fistulectomy)) [Title/Abstract]
3 (fistula-in-ano OR FIA) [Title/Abstract]
4 2 OR 3
5 1 OR 4
***Laser therapy terms*:**
6 "Laser Therapy"[Mesh]
7 "Photochemotherapy"[Mesh]
8 (laser OR fistula laser closure OR FiLaC OR photochemotherapy) [Title/Abstract]
9 6-8/OR
***Treatment outcome terms***
10 "Treatment Outcome"[Mesh]
11 "Postoperative Complications"[Mesh]
12 "Reoperation"[Mesh]
13 "Fecal Incontinence"[Mesh]
14 "Wound Healing"[Mesh]
15 "Recurrence"[Mesh]
16 "Surgical Wound Dehiscence"[Mesh]
17 (outcomes OR complication OR reoperation OR continence OR manometry OR healing OR recurrence OR dehiscence) [Title/Abstract]
18 10-17/OR
***Final search results*: *Combining Anal fistula and Laser therapy and Treatment outcome*:**
18 5 and 9 and 18

### Eligibility criteria

According to PRISMA guideline, the PICOS framework will be constructed in terms of population, interventions, comparisons, outcomes and study question or design to determine eligibility criteria. Studies satisfying the following inclusion criteria will be involved for the qualitative and quantitative analyses.

**Study Question**—Are the effectiveness, safety and outcomes of surgical laser treatment favorable and varied for cryptoglandular anal fistula stratified by Parks’ classification and different forms of laser?

**Study Design**—Interventional studies (randomized or nonrandomized clinical trials) or observational studies published in any languages will be eligible for inclusion;

**Populations**—Participants diagnosed with cryptoglandular anal fistula through clinical manifestations and/or endoanal ultrasound or magnetic resonance imaging (MRI) according to Parks’ classification (mainly involving intersphincteric, transsphincteric, suprasphincteric, and extrasphincteric fistulas).

**Intervention/exposure**—Laser ablation, fistula laser closure, photodynamic therapy or FiLaCTM treatment method as the primary intervention or exposure of interest. Based on the reported literatures and gain medium of lasers, we will classify them into gas lasers (including carbon dioxide [CO2] lasers), solid-state lasers (including the Nd:YAG laser) and semiconductor lasers or diode lasers. Moreover, if possible, we will also classify lasers by mode of operation including continuous-wave lasers and pulsed lasers.

**Comparators**—Studies will be included if they are single-arm or compared with patients not receiving laser ablation or FiLaC_TM_ treatment.

**Outcomes**—Studies will be included if any one of the reported primary outcomes are involved [24], which are classified as short-term outcomes including postoperative complications (including postoperative urinary retention, infection, incontinence, and bleeding that need interventions) and reinterventions which are assessed during hospitalization or one month after laser treatment; medium and long-term outcomes including success rate or recurrence rate, clinical fistula healing and radiological healing, development of additional fistulas, fistula symptoms, incontinence, psychological impact of treatment, patient satisfaction and quality of life which are assessed 3 to 6 months or one year after laser treatment.

In addition, in the presence of more than one paper of the available publications in laser fistula treatment from the same authors, all the publications will be reviewed and relevant information will be abstracted, but only the most recent or complete one will be selected.

### Data extraction

We will conduct data extraction on the characteristics of eligible studies from reports using a piloted, structured data extraction form by two independently authors. The results of the data abstraction will be further crosschecked by a senior author. All discrepancies will be resolved by mutual discussion. The structured data extraction form is composed by the following variables: name of the first author of the publication, study publication year, study design and set, original region the study conducted, time range of the participant enrolment, participant characteristics [(mean or median age at enrolment, gender, BMI of intervention measures, Parks classification of the disease, previous anal surgery history and comorbid disease (diabetes, autoimmune disease, or colitis)], intervention or exposure measures (laser parameters and type for FiLaC), comparison measures, criteria for ascertainment of anal fistula diagnosis and outcome ascertainment.

### Methodological quality assessment

For interventional studies such as RCTs, risk of bias will be assessed using the Cochrane risk of bias tool with the assessment of indicators of selection bias, performance bias, detection bias, attrition bias, and reporting bias [25]. For observational studies, we will use the Newcastle–Ottawa scale (NOS) tool to assess the study quality (risk of bias) [26]. A total of nine points will be awarded to each study in terms of the selection of the study groups, the comparability of the study groups and the ascertainment of the exposure and the outcomes. The NOS score ranges from zero to nine, and high-quality study is defined as a study with quality scores being more than 7 and moderate to low-quality study as no more than 7 [27].

### Grading of evidence

For RCTs, the Grading of Recommendations, Assessment, Development and Evaluation (GRADE) approach will also be conducted to rate the quality of evidence for each outcomehttps://www.ncbi.nlm.nih.gov/pmc/articles/PMC6949116/ - R18 [28]. Two authors will independently assess risk of bias, inconsistency of results, indirectness of evidence, imprecision, and publication bias. Overall quality is allocated very low, low, moderate, or high.

### Statistical analysis

All statistical analyses will be carried out with the software Stata 15.0 (Stata Corp LP, College Station, TX). Data from observational studies, RCTs and non-randomized studies will be analyzed separately. If the quantitative data are provided, a meta-analysis will be carried out for specific outcomes. If we are unable to pool data statistically using meta-analysis, a narrative synthesis of results adhering to the Synthesis Without Meta-analysis (SWiM) guideline will be conducted [29]. To assess the pooled treatment effects, we will measure relative risk (RR) and 95% CI for dichotomous outcomes [30]; while for continuous outcomes, we will combine weighted mean difference (WMD) (for the same outcome measurement) or standardized mean difference (SMD) (for different outcome measurement) and its corresponding 95% confidence intervals (CIs) [31]. We will use the Mantel Haenszel approach to pool dichotomous outcomes and the inverse variance method to pool continuous outcomes, respectively. We will examine between-study heterogeneity using the Cochrans Q-statistic and quantify the proportion of the total variation due to between-study heterogeneity using the I^2^ statistic. We consider an I^2^ > 50% representing substantial heterogeneity [32,33].

We will apply a random effects model, a more conservative approach to calculate the pooled estimates. Subgroup analysis (or meta-regression) based on the available variables will be performed to examine the potential factors contributing to between-study heterogeneity if there are sufficient data available for the study outcomes. Also, if possible, we will stratify the outcomes based on the fistula tract length to more than 3 cm and less than 3 cm from the anal verge. Publication bias will be assessed by inspection of funnel plot asymmetry and Egger’s regression test provided that 10 or more studies are included in the meta-analysis for the studied outcome [34]. Leave-one-out sensitivity analysis will be conducted to assess the robustness of pooled estimates. For all statistical analyses, a value of P < 0.05 will be deemed statistically significant.

### Ethics and dissemination

Ethical approval is not required as all the data we will be analyzed are obtained from published literatures. The results of our study will be disseminated in a peer-reviewed journal.

## Discussion

The benefit of laser treatment for cryptoglandular anal fistula is controversial. The recommendation of the application of this procedure for those patients is challenging due to the highly heterogeneous results of both interventional and observational studies conducted in these few decades.

Our study raises several concerns worth addressing: is laser therapy suitable for all types of anal fistula stratified by Parks’ classification? Is there any difference in the effectiveness of different forms of laser in the treatment of anal fistula? Whether there is any difference between short-term and long-term effects of laser therapy. Although there is more and more relevant evidence in recent years, it is obvious that no literature can fully address these concerns. Through critical systematic evaluation and quantitative synthesis of available data, we hope to give the most comprehensive answer to these questions so far.

The current study has a number of strengths in terms of the following aspects. Firstly, we will include the largest literatures in the relevant area, providing up-to-date evidence to evaluate the effectiveness, short-term and long term outcomes of laser treatment. Secondly, we will strict adherence to the Cochrane Library and PRISMA guidelines for systematic review and meta-analysis to conduct and report this study process. We have also developed systematic and comprehensive database search strategies for all the searched databases including PubMed, Embase, and Cochrane library without search date restriction in order to obtain relevant literatures as much as possible from all around the world, which may avoid the potential of publication bias on the pooled findings to a great extent, thus the repeatability of results could be much improved. Thirdly, we will conduct a transparent methodologic quality assessment of the included studies using either Cochrane tool for RCTs or the NOS checklist recommended for cohort studies. Fourth, we will also plan to use multiple approaches to test the potential heterogeneity, publication bias and the robustness of the results for each outcome, including subgroup analyses, sensitivity analyses, trim-and-fill analysis or meta-regression if possible.

## Conclusions

The real effectiveness, especially postoperative short-term and long-term outcomes of laser treatment on cryptoglandular anal fistula has not yet been determined. How to evaluate the exact effectiveness of laser treatment and the best indication for anal fistula surgery is the main purpose of this study. The synthesis of quantitative and qualitative evidence will yield updated and comprehensive evidence of laser treatment on specific outcomes, which can provide anorectal surgeons with high level evidence-based recommendations to improve patient care and clinical outcomes.

## Supporting information

S1 Table PRISMA-P checklist. (DOC)

## Abbreviations

APFCP: Asia Pacific Federation of Coloproctology
ASCRS: American Society of Colon and Rectal Surgeons
CI: confidence interval; Emtree
EMBASE: Subject Headings
ESCP: European Society of Coloproctology
GRADE: the Grading of Recommendations, Assessment, Development and Evaluation
LIFT: ligation of the intersphincteric fistula tract
MeSH: Medical Subject Heading
MRI: magnetic resonance imaging
NOS: Newcastle Ottawa Scale
PDT: photodynamic therapy
PRISMA-P: Preferred Reporting Items for Systematic Review and Meta-Analysis Protocols
RR: relative risk
SMD: standardized mean difference
WMD: weighted mean difference

## References

[1] Fugita FR, Santos CHMd, Ribeiro COdS. Epidemiological profile of patients with fistula in ano. Journal of Coloproctology (Rio de Janeiro). 2020;40:1–7.

[2] Włodarczyk M, Włodarczyk J, Sobolewska-Włodarczyk A, Trzciński R, Dziki Ł, Fichna J. Current concepts in the pathogenesis of cryptoglandular perianal fistula. Journal of International Medical Research. 2021;49(2):0300060520986669. doi: 10.1177/0300060520986669 . 33595349 PMC7894698

[3] Garg P, Sodhi SS, Garg N. Management of Complex Cryptoglandular Anal Fistula: Challenges and Solutions. Clinical and experimental gastroenterology. 2020;13:555–67. Epub 2020/11/19. doi: 10.2147/CEG.S198796 ; PubMed Central PMCID: PMC7667587. 33204136 PMC7667587

[4] Garg P. Transanal opening of intersphincteric space (TROPIS)—A new procedure to treat high complex anal fistula. International Journal of Surgery. 2017;40:130–4. doi: 10.1016/j.ijsu.2017.02.095 28259693

[5] Kang WH, Yang HK, Chang HJ, Ko YT, Yoo BE, Lim CH, et al. High ligation of the anal fistula tract by lateral approach: A prospective cohort study on a modification of the ligation of the intersphincteric fistula tract (LIFT) technique. International Journal of Surgery. 2018;60:9–14. doi: 10.1016/j.ijsu.2018.08.008 30343130

[6] Liu H, Tang X, Chang Y, Li A, Li Z, Xiao Y, et al. Comparison of surgical outcomes between video-assisted anal fistula treatment and fistulotomy plus seton for complex anal fistula: A propensity score matching analysis—Retrospective cohort study. International Journal of Surgery. 2020;75:99–104. doi: 10.1016/j.ijsu.2020.01.137 32014596

[7] Al Sebai OI, Ammar MS, Mohamed SH, El Balshy MA. Comparative study between intersphinecteric ligation of perianal fistula versus conventional fistulotomy with or without seton in the treatment of perianal fistula: A prospective randomized controlled trial. Annals of Medicine and Surgery. 2021;61:180–4. doi: 10.1016/j.amsu.2020.12.014 33489105 PMC7804334

[8] Garg P, Singh P. Video-Assisted Anal Fistula Treatment (VAAFT) in Cryptoglandular fistula-in-ano: A systematic review and proportional meta-analysis. International Journal of Surgery. 2017;46:85–91. doi: 10.1016/j.ijsu.2017.08.582 28882770

[9] Garcia-Arranz M, Garcia-Olmo D, Herreros MD, Gracia-Solana J, Guadalajara H, Portilla F, et al. Autologous adipose-derived stem cells for the treatment of complex cryptoglandular perianal fistula: A randomized clinical trial with long-term follow-up. Stem Cells Translational Medicine. 2020;9(3):295–301. doi: 10.1002/sctm.19-0271 31886629 PMC7031651

[10] Abdelnaby M, Emile S, El-Said M, Abdallah E, AbdelMawla A. Drained mucosal advancement flap versus rerouting Seton around the internal anal sphincter in treatment of high trans-sphincteric anal fistula: A randomized trial. International Journal of Surgery. 2019;72:198–203. doi: 10.1016/j.ijsu.2019.11.008 31751790

[11] Mei Z, Wang Q, Zhang Y, Liu P, Ge M, Du P, et al. Risk Factors for Recurrence after anal fistula surgery: A meta-analysis. International Journal of Surgery. 2019;69:153–64. doi: 10.1016/j.ijsu.2019.08.003 31400504

[12] Sofii I, Irianiwati Gunadi, Handaya AY, Fauzi AR. Combination of simple advancement flap and fistulectomy to treat complex anal fistula as a complication of hemorrhoidectomy: Case report. Annals of Medicine and Surgery. 2022;73:103203. doi: 10.1016/j.amsu.2021.103203 35028135 PMC8715064

[13] Garg P. Is fistulotomy still the gold standard in present era and is it highly underutilized?: An audit of 675 operated cases. International Journal of Surgery. 2018;56:26–30. doi: 10.1016/j.ijsu.2018.06.009 29886281

[14] Elfallal AH, Fathy M, Elbaz SA, Emile SH. Comprehensive literature review of the applications of surgical laser in benign anal conditions. Lasers in Medical Science. 2022. doi: 10.1007/s10103-022-03577-1 35606626

[15] Azadgoli B, Baker RY. Laser applications in surgery. Annals of Translational Medicine. 2016;4(23):1. doi: 10.21037/atm.2016.11.51 28090508 PMC5220034

[16] Lovat LB, Bown SG. Lasers in gastroenterology. World journal of gastroenterology. 2001;7(3):317–23. Epub 2002/01/31. doi: 10.3748/wjg.v7.i3.317 ; PubMed Central PMCID: PMC4688715. 11819783 PMC4688715

[17] Arroyo A, Moya P, Rodríguez-Prieto MA, Alcaide MJ, Aguilar MM, Bellón M, et al. Photodynamic therapy for the treatment of complex anal fistula. Techniques in coloproctology. 2017;21(2):149–53. Epub 2017/01/22. doi: 10.1007/s10151-016-1571-y . 28108825

[18] Arroyo A, Sánchez-Guillén L. Photodynamic Therapy for the Treatment of Complex Anal Fistula. 2020;52(6):503–8. doi: 10.1002/lsm.23162 . 31536149

[19] Brabender DE, Moran KL, Brady M, Carmichael JC, Mills S, Pigazzi A, et al. Assessing the effectiveness of laser fistulectomy for anal fistula: a retrospective cohort study. Techniques in coloproctology. 2020;24(10):1071–5. doi: 10.1007/s10151-020-02281-y 32770423

[20] Oztürk E, Gülcü B. Laser ablation of fistula tract: a sphincter-preserving method for treating fistula-in-ano. Diseases of the colon and rectum. 2014;57(3):360–4. Epub 2014/02/11. doi: 10.1097/DCR.0000000000000067 . 24509460

[21] Giamundo P, De Angelis M. Treatment of anal fistula with FiLaC(®): results of a 10-year experience with 175 patients. Techniques in coloproctology. 2021;25(8):941–8. Epub 2021/05/21. doi: 10.1007/s10151-021-02461-4 . 34013497

[22] de Bonnechose G, Lefevre JH, Aubert M, Lemarchand N, Fathallah N, Pommaret E, et al. Laser ablation of fistula tract (LAFT) and complex fistula-in-ano: "the ideal indication" is becoming clearer…. Techniques in coloproctology. 2020;24(7):695–701. Epub 2020/04/26. doi: 10.1007/s10151-020-02203-y . 32333136

[23] Shamseer L, Moher D, Clarke M, Ghersi D, Liberati A, Petticrew M, et al. Preferred reporting items for systematic review and meta-analysis protocols (PRISMA-P) 2015: elaboration and explanation. BMJ: British Medical Journal. 2015;349:g7647. doi: 10.1136/bmj.g7647 25555855

[24] Iqbal N, Machielsen AJHM, Kimman ML, Kane G, Woodcock R, Grossi U, et al. AFCOS: The Development of a Cryptoglandular Anal Fistula Core Outcome Set. Ann Surg. 2022 Jul 11. doi: 10.1097/SLA.0000000000005462 Epub ahead of print. . 35815887 PMC10082062

[25] Higgins JPT, Thomas J, Chandler J, Cumpston M, Li T, Page MJ, et al. Cochrane Handbook for Systematic Reviews of Interventions version 6.3 (updated February 2022). Cochrane, 2022. Available from www.training.cochrane.org/handbook.

[26] Stang A. Critical evaluation of the Newcastle-Ottawa scale for the assessment of the quality of nonrandomized studies in meta-analyses. European journal of epidemiology. 2010;25(9):603–5. Epub 2010/07/24. doi: 10.1007/s10654-010-9491-z . 20652370

[27] Li C, Xiao Y, Hu J, Hu Z, Yan J, Zhou Z, et al. Associations Between Diabetes and Idiopathic Pulmonary Fibrosis: a Study-level Pooled Analysis of 26 Million People. J Clin Endocrinol Metab. 2021;106(11):3367–3380. doi: 10.1210/clinem/dgab553 . 34302736

[28] Guyatt G, Oxman AD, Akl EA, Kunz R, Vist G, Brozek J, et al. GRADE guidelines: 1. Introduction-GRADE evidence profiles and summary of findings tables. Journal of clinical epidemiology. 2011;64(4):383–94. Epub 2011/01/05. doi: 10.1016/j.jclinepi.2010.04.026 . 21195583

[29] Campbell M, McKenzie JE, Sowden A, Katikireddi SV, Brennan SE, Ellis S, et al. Synthesis without meta-analysis (SWiM) in systematic reviews: reporting guideline. BMJ. 2020;368:l6890. doi: 10.1136/bmj.l6890 ; PMCID: PMC7190266. 31948937 PMC7190266

[30] Schwarzer G, Carpenter JR, Rücker G. Meta-analysis with binary outcomes. Meta-analysis with R: Springer; 2015. p. 55–83.

[31] Riley RD, Lambert PC, Staessen JA, Wang J, Gueyffier F, Thijs L, et al. Meta-analysis of continuous outcomes combining individual patient data and aggregate data. Statistics in medicine. 2008;27(11):1870–93. Epub 2007/12/12. doi: 10.1002/sim.3165 . 18069721

[32] Higgins JP, Thompson SG, Deeks JJ, Altman DG. Measuring inconsistency in meta-analyses. BMJ (Clinical research ed). 2003;327(7414):557–60. Epub 2003/09/06. doi: 10.1136/bmj.327.7414.557 ; PubMed Central PMCID: PMC192859. 12958120 PMC192859

[33] Higgins JP, Thompson SG. Quantifying heterogeneity in a meta-analysis. Statistics in medicine. 2002;21(11):1539–58. Epub 2002/07/12. doi: 10.1002/sim.1186 . 12111919

[34] Egger M, Davey Smith G, Schneider M, Minder C. Bias in meta-analysis detected by a simple, graphical test. BMJ (Clinical research ed). 1997;315(7109):629–34. Epub 1997/10/06. doi: 10.1136/bmj.315.7109.629 ; PubMed Central PMCID: PMC2127453. 9310563 PMC2127453

